# Cognitive Capacities as Functional Natural Kinds

**DOI:** 10.1007/s12124-024-09863-7

**Published:** 2024-08-26

**Authors:** Claudia-Lorena García, Mariana Salcedo-Gómez, Alejandro Vázquez-del-Mercado

**Affiliations:** 1 Instituto de Investigaciones Filosóficas, Universidad Nacional Autónoma de México, CDMX, México; 2 División de Ciencias Sociales y Humanidades, Universidad Autónoma Metropolitana, CDMX, México; 3 Facultad de Filosofía y Letras, Universidad Nacional Autónoma de México, CDMX, México

**Keywords:** Cognitive explanation, Interventionist causation, Neurocognitive integration, Cognitive variation, Multiple realization, Individuation of cognitive capacities, Mechanisms

## Abstract

In this paper, we articulate a functional approach to cognitive capacities. It is a *restricted functionalism* for various reasons, but especially because it does not claim that all cognitive (and/or mental) entities and processes are functional in the sense of a systemic capacities approach. One of the central aims of a cognitive theory consists in providing explanations of behavioral phenomena of (human and non-human) animals, and of the phenomena that are involved in those explanations. We accept that part of what lies at the heart of these explanations are certain functional entities –we call them “cognitive functional systems” –which in our view stand for most of the cognitive capacities of an organism; that is, systems that are individuated primarily by the main cognitive functions they undertake. Additionally, in the paper, we go into further detail concerning these functional systems, their internal organization, the nature of their causal interactions, etc. We also argue that some of these classes of cognitive functional systems (i.e., cognitive capacities) can be construed as “*natural kinds*” whenever their kinds of functional organizations are understood as kinds of hierarchically ordered classes of information processing events that are related among each other in regular (often complex) ways.

## Introduction

The status of cognitive kinds has been the subject of a lively debate since the second half of the 20th century. In the last decade, this topic has received renewed interest in the philosophy of cognitive science. In the present section, we give a general context for our proposal concerning cognitive kinds and offer a general outline of some of its theoretical motivations.

Initially, the perspective on these kinds in philosophy was highly influenced by the classic computationalism view, related to the Symbolic Systems Hypothesis (Simon & Newell, 1976). Under the computationalism framework, the nature of human cognition was assumed to consist of symbolic manipulation, especially logic, within something in the line of a Von Neumann architecture. In this paradigm, implementation was deemed irrelevant, and cognitive explanations were thought to be found at the algorithmic level (Bermúdez, 2014). Within philosophy of mind, machine functionalism (e.g., Putnam 1960) was widely adopted as a metaphysics, though other types of functionalism less closely aligned with symbolic processing followed. The notion of interest was that of a “mental process.” Functionalism in this sense, defines a mental process or capacity as a function (in the mathematical sense) that has as an input external sensory stimulation and/or other mental states, and as an output observable behavior and/or other mental states. In this context, philosophical arguments were offered to justify the autonomy of psychological laws (Fodor, 1980).

Nonetheless, the symbolic paradigm was not entirely representative of all the existing work in cognitive psychology at the time, which often dealt with information processing models in broad terms without a particular commitment to a general architecture of cognition or to symbolic processing. What has been representative of cognitive psychology –symbolic or not– were functional explanations in a broader sense; that is, the description of information processing understood in the broad manner in which functional systems à la Cummins (2000) with their subsystems (and the subsystems of these subsystems, and so on) and a description of their activities (or functions) and interactions among them.^1^ Without abandoning functional explanations, psychology has increasingly moved towards exploring other types of structures such as heuristics, Bayesian modeling and connectionist networks.

Within the philosophy of psychology, this was accompanied by a growing consensus that psychology did not consist in the search for autonomous “laws”, but in the description of cognitive capacities having different cognitive functions (Cummins, 2000) such as working memory, episodic memory, reasoning, attention in various forms, etc. It should also be noted that models in cognitive psychology are not generally committed to folk or intentional taxonomies, such as “belief” or “desire”. Instead, cognitive capacities are often couched in terms of encoding, comparing, storing, and retrieving information (Shapiro, 2017, p. 1056).^2^

These functional models of cognitive capacities are meticulously built by generating experimental settings and analyzing its results. Cognitive tasks are given in tests to obtain valuable data, for example about the ability of subjects to do tasks and the processing time it takes, which allows to build models which individuate capacities by their regular activities and contributions to cognition. This style of model building is especially done within cognitive psychology. It has provided predictive and explanatory models in areas such as memory (episodic, working, long-term) and linguistic processing (sentence parsing, lexical comprehension, etc.).

Nevertheless, without the constraints of the old computationalist paradigm, there are no agreed ontological constraints on the organization of the cognitive processes and the entities that underlie these capacities beyond their empirical adequacy (regarding the prediction of tasks, processing times, etc.)—neither there seemed to be agreement on the degree to which one has to take into account data from other disciplines such as neuroscience, biology and psychiatry.

In our view, the most important ontological constraints derive from two considerations. First, the requirement is that anything studied in cognitive science will, sooner or later, be causally and/or functionally connected with the production of relevant behavior in the organism under study. The behavior in question is usually typified and understood, not as the sheer precise physical (i.e., physiological, anatomical) movements of an animal’s body, but principally in terms of the functions that it has in connection with its environment. Second, and related to the previous point, the main cognitive capacities postulated in cognitive science must be conceptualized as cognitive functional systems,. i.e., as systems whose function is, to put it roughly, to transform information or representations that ultimately and jointly resolve into functionally described behavior.

Our concern is that mere empirical adequacy is not enough. This is where our proposal concerning which cognitive capacities can be considered as natural kinds comes into play. For realists about the pursuits of cognitive science, such as ourselves, there is a difference between models that are merely predictive and those that aim to represent the actual structure of human cognition. Of course, cognitive psychologists use implicit criteria in choosing the capacities that are suitable for building models. Still, there is more work to be done regarding the elaboration of an explicit and philosophically sound metaphysics of cognitive kinds.

However, the continuous actual development of cognitive neuroscience further complicates the picture, since its relation to cognitive psychology has been a source of controversy and confusion. This has been due in part to the fact that much of the work in that discipline has assumed that there must be a strong reductionism between the cognitive and the neural. Unsurprisingly, the complexity of the controversy surrounding the relations between psychology and neuroscience has become a subject in the philosophy of cognitive science. Thus, the scientific status of functional psychological models has recently become the subject of an increasingly technical debate within the philosophy of cognitive science. This debate is generally related to the discussion concerning the extent to which cognitive phenomena can be said to be in a straightforward relation to neural processes (see for example the articles respectively collected in Kaplan, 2017b, and Calzavarini & Viola, 2021). As we shall see, the neural capacities postulated in various accepted pursuits are within the scope of the functional cognitive study we are proposing, although none of them are in principle excluded as additional parts that contain additional information useful to the functional picture of cognition.^3^

Presently, in the philosophy of neuroscience there are at least two important issues that we will examine here:

1. Is psychology autonomous from neuroscience? should all psychological explanations be integrated with neuroscientific explanations?
2. How do these two different types of research (psychological and neural) constrain each other?

There are two positions in this debate: the mechanistic position and the functionalist position. The former asserts that genuine explanations have to postulate mechanisms which are systems that produce regular activity through the interaction of its parts, where the parts are spatiotemporally described entities usually at the level of neuroscience (e.g. Craver, 2009, Boone & Piccinini, 2016). Functionalists also aim to characterize explanations as systemic, but they contend the system is functionally individuated regardless of mechanical constitution, and we agree with them on this point. In contrast, other functionalists tend to argue that psychology is taxonomically and also explanatorily autonomous from neuroscience (e.g. Weiskopf, 2017, Roth & Cummins, 2017). In Sect. “Cognitive Natural Kinds, Individual Variation, and Multiple Realization” we argue for the taxonomical autonomy, but reject a form of explanatory autonomy.

One last point of clarification. There are many functionalist theories that have been proposed by philosophers, psychologists and cognitive neuroscientists. Yet our functionalism differs from most of them in one or more of the following points:

- The ontological status of some cognitive capacities or CF-systems has to be considered seriously (what philosophers call ‘natural kinds’). Concerning this point, researchers like Craver (2009), Piccinini and Craver (2011), and Cummins (2000) (although their theories are heterogeneous) seem to reject the idea that cognitive entities like functional systems (or mechanistic systems, in the case of Piccinini and Craver) are ontologically sound on their own. In contrast, in our view, there are some classes or kinds of cognitive functional systems (i.e., cognitive capacities) that, in a technical sense to be explained, can be considered as *natural kinds*.^4^
- We introduce a distinction between cognitively simple and cognitively complex functional systems (see Sect. “Cognitive Functions, Functional Explanation, and CF-Systems” below). This distinction is a part of the explanation of the interface between neuroscience and cognitive science. As far as we can tell, a clear-cut distinction between cognitively simple and cognitively complex CF-systems has not yet appeared in the writings of other researchers beforehand.
- One of the central ideas around which our view is articulated (see Sect. “Cognitive Functions, Functional Explanation, and CF-Systems”) is that of facilitating the possibility that most of the functional organizations of the cognitive systems of humans and non humans derive with modifications from the cognitive organizations of systems of the last common ancestors of the relevant groups in a lineage (in a way that one should be able to reconstruct their cognitive evolutionary history). The distinction between simple and complex CF-systems can also help explain both the possibility of evolutionary novelties in the cognitive realm and cognitive evolution in general.
- Our paper also contains a discussion concerning multiple realization. And this touches upon questions concerning the interface between cognition and neural processes. Our position in this respect is that the cognitive taxonomy of CF-systems is autonomous from neuroscientific taxonomies. In relation to this idea, we oppose the views defended by, e.g., Piccinini and Craver (2011). However, in a sense to be explained below (Sects. “Cognitive Natural Kinds, Individual Variation, and Multiple Realization” and “Functionalism, Mechanism, and the Neuro-Cognitive Debate”), we argue that the explanation of cognitive capacities is probably not completely independent of neurological explanations. Regarding these points, we oppose on the one hand, the views of Piccinini and Craver, and on the other, the view of Weiskopf (2017), who argues for complete explanatory independence of cognitive psychology from neuroscience.
- Finally, as we have already mentioned, our functionalism is restricted, since it only claims that cognitive capacities are functional systems. We make no functionalist assertion concerning other cognitive entities. This is also a distinguishing feature of our view.

## Desiderata for an Acceptable View of Cognitive Capacities and Their Natural Kinds

Our first step consists in stating the main desiderata that we think a global view on the ontology and taxonomy of cognitive capacities should fulfill. As we later articulate the different parts of our view, we shall indicate how or why these desiderata are fulfilled (whenever this is not obvious).

- Methodological adequacy: it should be faithful to scientific practice, with a special emphasis on cognitive psychology and cognitive neuroscience. Philosophically motivated revisionism is to be avoided.
- Nomological underpinnings: the processes and entities that are postulated in our view of natural kinds must appeal to the existence of nomological regularities that constitute the cognitive dispositions that are part of those capacities and entities.^5^
- The postulated cognitive capacities are sufficiently stable dispositions, within the boundaries of certain manageable contexts, as to allow some form of causality (interventionist, in our case) to be applicable.
- It should allow for different levels of complexity: some cognitive capacities are simpler, such as edge detection, while others are more complex such as face recognition. Usually, the former is involved in the formation of the latter.
- Cognitive capacities may be studied by a variety of methods, and at different levels. Some phenomena are studied at the level of neural mechanisms, some at the level of systems neuroscience, and others at the functional level of explanation, such as a great part of cognitive psychology. However, the picture concerning the relationships between the methods of these different disciplines is fairly complex.
- It must allow for the interaction and often nested organization of cognitive capacities. For example, the phonological loop is a cognitive capacity which in turn forms part of working memory (a higher capacity).
- It should be neutral with respect to the nature of a cognitive capacity’s neural implementation. For example, to what degree a cognitive capacity is computational, as well as implemented by neural networks or dynamic patterns, is an empirical matter; all that must happen is that there is a central functional description of that capacity in terms of the production of an informational or representational output.
- It should also be neutral with respect to multiple realization and intertheoretical reduction, which also are entirely a posteriori matters to be empirically decided in the long run.

With these desiderata in mind, we develop the notions of a cognitive capacity (or CF-system) and of a cognitive functional natural kind or CF-kind. It is important to emphasize that achieving our objective does not consist in simply showing that cognitive kinds are natural by taking a previous notion of natural kind and then adopting a taxonomy that fits our view of cognitive capacities ad hoc. This is not possible, since, as the debate between mechanists and functionalists shows, there is no agreement regarding the correct taxonomy. Instead, we aim to show how minimal realist presuppositions about cognitive kinds and attention to scientific practice are captured by the notion we propose.

It should be noted that our work does not aim to prove that all the cognitive capacities postulated in cognitive science to date are natural kinds. This could only be done on a case-by-case basis, by considering the empirical evidence for the ontological robustness of each capacity or a selected number of them. It is even possible that, when all is said and done, no cognitive capacities are natural kinds; say, if the cognitive dispositions of each and every species turn out to be holistically organized beyond the point of any possible decomposition (which is extremely unlikely from the presently available evidence). Instead, we take the existence of ontologically robust, distinct cognitive capacities as a given and work out an appropriate notion of natural kind. This point is widely shared by competing views on cognitive ontology in the context of the debate mentioned in the previous section. We argue for the specific points of our proposal appealing to scientific practice and minimally realist concerns, remaining neutral on whether the notion we propose is useful or appropriate for other domains where functional taxonomies are highly prevalent (e.g. biology).

In the next section, we explain our notion of a cognitive-functional system and related notions such as that of function, functional explanation, cognitive simplicity in opposition to complexity, etc. We also present the kinds of cognitive capacities that we have in mind as paradigmatic examples of capacities that can be articulated as CF-systems.

## Cognitive Functions, Functional Explanation, and CF-Systems

The core idea of functionalism is that there were functional entities that were individuated, picked out, explained, and/or organized in terms of the psychological function(s) they undertook. The functions in question were ultimately understood in terms of the generation (in the relevant organisms) of certain kinds of behaviors. Cognition then was understood as involving entities which (directly or indirectly, overtly or covertly) caused and/or explained the behavior of animals –mainly, human animals. The behavior itself was usually typified not as the sheer precise physical movements of an animal’s body, but rather in terms of the effects that they had on their immediate environment (and sometimes on the generation of other internal states). In the particular case of the behavior of non-human animals, one of these effects –its function– was usually understood as the biological “function” of that behavior-falling under types such as feeding, mating, parenting, migrating, predating, etc. (Millikan, 2020; Neander, 2017). These are the features which our interventionist view of the nature of cognitive functional systems picks out as the variables under intervention.

There is nowadays a wide consensus concerning the manner in which functional analysis, functional systems, and a systemic notion of function ought to be conceptualized.^6^ Functional analysis consists of the decomposition of an entity into its functional parts and the manner of their interaction, and of these parts into their functional parts, and so on;^7^ these procedures allow us to uncover the functional organization of that entity, and the end result is what is called a *functional system*. Cummins (1975) uses the notion of functional analysis to characterize a notion of function, usually called “a systemic notion of function”. The main idea here is that the function of a component is the contribution it makes to the operation of a larger system of which that component is a part.^8^ Furthermore, the activity *A* of a system *S* is functionally explained when it is shown how it is composed, the functions of its parts, and the manner in which the parts interact causally to produce the activity in question. Here we accept the view that some cognitive capacities are functional systems in this broad sense,^9^ though we shall diverge in important ways from existing proposals in this tradition –most notably Roth and Cummins (2017), and Weiskopf (2017)– since we require further ontological constraints.^10^

Additionally, a functional system can be composed of parts that are themselves functional systems, that is, systems that accomplish function A by having a number of components which interact with each other, and which undertake each its own function. Thus, functional systems may have a nested structure: a system that has subsystems each of which in turn have other subsystems and so on. More needs to be said concerning functional systems, as we articulate them:

- They may be complex entities that are hierarchically interrelated.
- They are causal probabilistic structures – where causality is understood as Woodward (2013) does.
- They are correctly describable as sets of interventionist counterfactual statements.

For the sake of brevity, we will refer to cognitive functional systems as “CF-systems” –i.e., systems whose individuating function is cognitive. Also note that, in our view, a CF-system is a cognitive capacity that is functionally described, in the sense explained above. Another important point to emphasize here is that we assume a version of the idea that there is a way to capture a notion of *levels of description* that is coherent, conceptually fruitful, and actually in use in the special sciences (García, manuscript). We do not rely on any particular construal of this notion. We assume it because, as we shall see, it is necessary to articulate some of the ideas that are central to our view, e.g., the idea that some kinds of cognitive capacities may be multiply realized at the neurobiological level, and the idea that there are cognitively simple as opposed to cognitively complex CF-systems.

Let us now turn to CF-systems. When one describes a CF-system and its parts at the cognitive level, we will say that these parts are CF-subsystems of that CF-system. On the other hand, when the parts of a CF-system belong to a lower level (say the neuro-cerebral level), we will say that these parts *implement or realize* the CF-system.^11^ For example, the human capacity of working memory is conceived by some psychologists as a CF-system that is composed of a number of *cognitive* parts or subsystems: (a) The *phonological loop*, which receives auditory input and keeps it for a short period of time in (b) the *episodic buffer*, in which visuospatial representations are also temporarily stored after being received and (sometimes) rehearsed by (c) the *visuospatial sketchpad*; and most of these activities are controlled by (d) a *central executive* which consists of a supervisory activating system (Baddeley, 2003).^12^ In this case, we would say that human working memory is composed of those CF-subsystems, and the functions and causal interactions of these subsystems are *cognitively described* however, not yet in terms of neurons, cell groups, kinds of tissue, brain regions, and their dynamic interactions. These cognitive subsystems –for example, the phonological loop– may or may not be cognitively analyzable as being composed of yet other simpler cognitive subsystems. If they are cognitively analyzable, then their cognitive parts and their interactions will have to be further investigated in cognitive science. If they are not, then (as a kind) they may be uniquely or multiply realized in the brain. And the brain regions and cell groups where the cognitive function of, say, the phonological loop are undertaken are said to (uniquely or multiply) *implement* its function, that is, the function may be implemented in the same or in distinct types of brain cell groups in different individuals.^13^ Although we want to say that *token* CF-systems of the same cognitive kind have a spatial localization in the brain and/or body of an organism, we do not assume that this localization is necessarily of the same neuro-cerebral and/or bodily *kind* for all these tokens. For instance, face recognition in a human population may be individually implemented with lesser or greater localization differences (García, 2013).

With these ideas in mind, we can now introduce a distinction between *simple* and *complex* CF-systems: A token CF-system is cognitively *simple* if and only if it is nomologically impossible to functionally analyze it any further into *cognitive* CF-subsystems (each of which has a cognitive function). On the other hand, a *complex* CF-system consists of a set of CF-subsystems, and a cognitive functional organization, i.e., by a class of regular causal interactions among its CF-subsystems. This does not mean that *simple* CF-systems cannot be functionally analyzed (in a broad Cumminsean sense of functional analysis); but its analyses will belong to lower levels of organization (e.g. the neural level).

Actual plausible candidates of *simple* CF-systems may be found in heterogeneously color-tuned neurons in macaque vision (Conway, 2002). This type of specialized neuron is not cognitively simple, since its receptive field can be decomposed into multiple subregions tuned to different colors (Nigam et al., 2021). These subregions encode color information, which itself is part of a larger, complex color encoding process. In this example, we would consider the receptive field of such neural subregions as cognitively simple.

Another –possibly more controversial– example of simple CF-systems are the so-called *classical* mirror neurons, each of which is activated by a visual and a motor stimulus (stimuli coming from the visual and the motor cortices) associated with a very specific type of action (e.g., grabbing a specific type of object in a certain manner). Each of these neurons has a cognitive function, which is to correlate sensory and motor information concerning the same “action” (see, e.g., Rizzolatti & Craighero, 2004). They probably are cognitively simple in our sense (i.e., they cannot be said to be composed of cognitive parts), yet they are probably cognitive parts of other more complex CF-capacities such as the imitation of observable actions of other members of one’s social group.^14^

Note that CF-systems (like many other kinds of natural functional systems) tend to have certain key characteristics worth mentioning. First, when and if they are part of a larger complex system (in our case, the cognitive system as a whole), then they would tend to be *combinable* –i.e., they would tend to combine in organizationally coherent ways at the brute-causal or at the cognitive-functional level to form CF-systems that are more complex (Wimsatt, 2013). Another interesting characteristic that functional systems tend to have is that of *functional integration* (Wagner & Schwenk, 2000). This characteristic often works as an evolutionary constraint on the types of non-lethal functional organizations that are evolutionarily available in a functional architecture at a certain time. Let us now briefly simply point towards one additional advantage of our view, which inextricably includes a distinction between *simple and complex* CF-systems. This distinction allows us to articulate further ideas that are at the interface between cognitive science and evolutionary biology. We assume, as most evolutionary biologists do, that evolution is parsimonious (i.e., that it tends to reuse, whenever possible, the resources that already exist in a lineage in order to find more advantageous ways to solve an adaptive problem).^15^

Oftentimes, tokens of two or more simple CF-systems already existing in a lineage of relatively simple organisms tend to causally combine in ways that leave their internal functional integrity mostly intact: the cognitive functions of each are the same as before as well as (much of) their internal lower-level organization (Wagner & Schwenk, 2000).^16^ The causal-functional combination of two or more simple CF-systems can constitute a complex CF-system having a novel cognitive function that may be evolutionarily advantageous to the organism possessing it; and if this complex CF-system turns out to be heritable, then it may be naturally selected for in the end and become a part of the CF-phenotype of the members of a species leading to a novel kind of cognitive capacity arising in evolutionary time. Of course, this may not be the only manner in which CF-novelties may emerge in evolution. Tokens of new simple CF-systems may have arisen later in the lineage, as a result of changes in the lower-level neural circuitry. Further, a simple CF-system may substitute another one in an already complex CF-system, making the complex CF-system acquire an additional C-function, or modifying the original complex C-function. Two relatively complex CF-systems may causally combine in ways that make for more interesting and stronger CF-capacities, and so on.

Since, in this context, the notion of evolvability and that of variation are closely related, we will need to make use of notions of *functional variation* and (as García (2010) explains) of functional homology to be able to speak of the evolvability of a cognitive capacity (understood as a CF-system). With functional homology, we mean the presence of the same kind of functional system in a lineage, due to descendance from the last common ancestor. 17

Thus, the simple-complex distinction concerning CF-systems is crucial to explain how cognition can be a complex phenomenon that nonetheless has a high degree of evolvability –a complexity that can be explained in a non-reductive manner by reference to lower-level descriptions. We now turn to explain what we mean by “natural kind” (NK) when we say that *some CF-systems are NKs*.

## Cognitive Functional Natural Kinds (CF-NK)

There appears to be a consensus among philosophers of science on the thesis that the traditional notion of natural kind –as a class of necessary and sufficient properties constitutive of the kind –does not apply to the categories and concepts that are postulated by the special sciences, like biology and cognitive science. If, to this thesis, one adds the widely held view that one can grant a category a privileged ontological status only when the category in question can *reasonably* be considered a “natural kind” –then it would turn out that the categories and concepts of the special sciences are theoretically weak and are at most heuristic devices that may prove helpful to us in moving around the world but of no great scientific interest.

Many philosophers for different but good reasons are dissatisfied with this consequence. They appreciate that scientists of disciplines of the special sciences have fashioned a great number of theoretically fertile and strong categories and concepts. These concepts would indeed fit what developmental biologists, biological anatomists and physiologists, neurologists as well as psychologists, cognitive scientists, cognitive ethologists and many others, consider as standing for explanatory, causal, projectible, inductive and deductive kinds usable in their scientific endeavors. Here we find the proposals of many *philosophers* of science (e.g. Kornblith, 2002; Boyd, 1999a, 2010; Khalidi, 2015 to mention a few) who attempt to fashion notions of natural kind different from the traditional notions that, in their views, may have as a result that some or most of those fertile concepts stand for natural kinds.

As we will soon be able to appreciate, the natural kind notion that we hereby propose bears some “family resemblance” to some of the aforementioned notions, but it sharply differs from them in some respects. Intuitively, a cognitive NK is a class of cognitive capacities (understood as CF-systems) that is referred to by some term belonging to a cognitive discipline that has various theoretic virtues. It is also a class whose members are cognitive capacities that may be cognitively very complex in a causal sense. In particular, we will say that a class *C* of complex CF-systems is an NK only when all token CF-systems belonging to kind *C* have the same cognitive function *Φ*; each token CF-system *S* of *C* is such that if some of its CF-subsystems tend to be robustly correlated in a significant number of cases, this robust correlation is attributed to a set of probabilistic nomological regularities regarding the manner in which the occurrence of some of its CF-subsystems tends to causally favor the occurrence of many others also in *S*. Further, it is nomologically possible that not all token complex CF-systems of NK are constituted by exactly the same kinds of CF-subsystems.^18^ A bit more precisely, we will say that a class of CF-systems constitutes a *natural kind* CF-NK only when:

A. All of the token CF-systems in the class CF-NK have the same cognitive function *Φ*.
B. All of the token CF-systems in CF-NK are either cognitively simple (in which case they are implemented at a lower level of organization), or cognitively complex (i.e., each of them contains as parts more than one cognitively simple or complex CF-subsystems, *S*_*1*_… *S*_*n*_).^19^
C. If CF-NK contains *simple* CF-systems, their function *Φ* being to produce certain kind of cognitive result, then for each token simple CF-system of this kind we will say that reference to its token lower-level implementation correctly or adequately explains (perhaps, mechanistically) how the outcome in question *tends* to be produced.^20^
D. If CF-NK contains *complex* CF-systems, then for each such token complex system *S* of CF-NK, *S* is composed of tokens of more than one kind of (simple or complex) CF-systems, let us call them *S*_*1*_,…, *S*_*n*_, *such that each of the S*_*i*_ (*i = 1*,*…*,* n) is an interactionist causal factor* (correctly describable by a counterfactual probabilistic conditional of the sort that Woodward (2013, 2021) proposes as descriptive of the occurrence of an interventionist probabilistic cause) –a causal factor of the cognitive outcome that *S tends* to produce and that is S’s function to obtain. Most of the *S*_*i*_ that compose the complex system *S* of CF-NK are such that they typically (albeit not necessarily) are causally responsible for the production of the outcome *Φ* that is *S*’s function, but there may be rare cases where the *S*_i_ are causally organized differently in the production of *Φ*.^21^
E. There is a set of nomological probabilistic dispositions regarding how the *S*_*i*_ of *S* tend to fulfill their own distinct functions, and how these nomological regular tendencies causally interact in order to form a complex probabilistic organization describable by a set of related correct interventionist counterfactual conditional probabilistic statements concerning how *S*’s subsystems are disposed to causally interact with each other in the production of *Φ*.^22^

As we can now appreciate, a cognitive natural kind, as we articulate it, is a class of cognitive capacities, and the corresponding notion has the virtue of allowing the members of the kind to be subject to variation and multiple realization.^23^ This is because –to put it in a simpler and less exact manner– either simple or complex cognitive-functional natural kinds are characterized as being classes of cognitive capacities (or CF-systems) that are themselves composed of entities that have certain causal *tendencies* describable in *counterfactual probabilistic* terms. This allows for certain strong forms of variation, such as those found in compensatory cognitive processes in alternative developmental pathways (e.g. due to neuroplasticity). Additionally, one can also appreciate that the question as to whether a class of CF-systems constitutes an NK is a theoretical question that must be answered in an *a posteriori*, rather than an *a priori* conceptual manner.

It is important to note that an interventionist causal notion (such as Woodward’s) applies only to certain sorts of causal arrangements.^24^ For example, he argues (2013, p. 46) that certain mechanistic structures that have been described recently in the philosophical literature on mechanism and mechanistic explanations (e.g. Bechtel, 2007; Craver & Tabery, 2015; Piccinini & Craver, 2011; Kaplan, 2017a, 2017b) seem to fulfill the conditions he identifies as important for the applicability of his interventionist concept of causation to certain causal structures; namely, stability, modularity (which he describes as another form of stability), organizational sensitivity or fine-tunedness. The extent to which an interventionist notion of causation is applicable to all the classes of cognitive capacities that turn out to be natural kinds (as we characterize them) is an *a posteriori* matter to be decided on a case-by-case basis. Yet we see no serious in principle obstacle to its applicability.^25^

Note that an important theoretical connection to our view concerning natural kinds can be found in Khalidi’s ideas of natural kinds as nodes in causal networks (2015), where we find a view of such kinds in a Boydean spirit.^26^ He explicitly excludes the homeostatic mechanisms posited by Boyd (1999b) as unnecessarily restrictive –for instance, it does not seem to apply to biological species– and as something obscuring their central aspect (Khalidi, 2015, 1386). Regarding this point, we agree with him. Thus, Khalidi (2015) characterizes natural kinds as hierarchically ordered, highly connected nodes in causal networks.

In particular, in connection with cognitive science, he examines some cognitive notions, such as domain specificity and innateness, that are not themselves capacities but characteristics of capacities (Khalidi, 2023). He also examines concepts of cognitive capacities, such as the capacities of concept formation, structuring and transformation in cognitive psychology, the capacity of episodic memory, some language-thought capacities, and heuristic capacities. However, in examining the variety of entities postulated in cognitive science –not only cognitive capacities–it is far from clear what he means in each case as a “node in a causal network” beyond the vague idea that such entities enter into a number of causal interactions of various sorts. In particular, in connection with cognitive science, he examines some cognitive notions, such as domain specificity and innateness, that are not themselves capacities but characteristics of capacities (Khalidi, 2023). He also examines concepts of cognitive capacities, such as the capacities of concept formation, structuring and transformation in cognitive psychology, the capacity of episodic memory, some language-thought capacities, and heuristic capacities. However, in examining the variety of entities postulated in cognitive science –not only cognitive capacities–it is far from clear what he means in each case as a “node in a causal network” beyond the vague idea that such entities enter into a number of causal interactions of various sorts.

There is a problem for Khalidi concerning how to draw the boundaries of the entities and interactions that enter, and those that do not enter, into the complex causal network that constitutes a causal node. To understand this problem, it would be useful to look at how we solve this problem in our systemic functional approach to cognitive capacities. We assert that a capacity is picked out (i.e., individuated) by a main cognitive function—this function, depending on context, may be biological in Millikan’s sense (e.g., in cognitively simple CF-systems), and/or Cumminsean systemic (in simple and complex CF-systems). The reasons to pick out a certain cognitively simple or complex system as something to be explained is not an arbitrary matter, as it is not arbitrary the choice of entities, information transforming processes, and interactions among these entities that are relevant parts of the CF-system in question. The main function of the CF-system dictates these choices—it is the typical outcome of a well-functioning CF-system belonging to the relevant natural kind. Without reference to a cognitive function, then, any of the “cognitive activities” –as Craver, Piccinini, Khalidi, etc. call them—of all the innumerable “systems” we have in front of us could as well be chosen, and no choice will seem to be arbitrary.^27^ Indeed, not every “activity” can be thought of as a function, in either the Millikean or the Cumminsean senses, and in our view the cognitive functions that can be chosen as the main individuating cognitive functions of a relatively robust kind of CF-system are those that fulfill the conditions enumerated above for cognitive functions *natural kinds*.

Thus, although we consider Khalidi’s ideas (2013; 2023) in this respect a very interesting attempt at articulating a metaphysically and methodologically sound notion of natural kinds for cognitive science, we think that a more detailed discussion of the aforementioned problems of Khalidi’s characterization of complex organizational nodes is a critical matter that cannot be undertaken here and yet deserves our full attention elsewhere.^28^

We now proceed to characterize and detail some of the *epistemic* features which, in our view, the cognitive disciplines that postulate CF-NK-*terms* typically have:

- They point to the existence of certain nomological regularities in the manner in which the CF-systems of a kind are instantiated by certain CF-subsystems –a set of regularities that is also constitutive of the kind term. This makes it probable that the term referring to a genuine NK of a certain cognitive discipline supports many strong inductive and abductive inferences, and sound deductive inferences, and certain explanatory processes that are correct by the standards of the cognitive discipline to which the theoretical NK term belongs.
- Cognitive NKs are always postulated in a certain cognitive disciplinary context, and the terms that refer to them there usually have many epistemic explanatory, inductive, and abductive connections with other terms of NK also appearing in that disciplinary context, in a way that the statements containing those NK-terms tend to form strong and complex epistemic networks.
- The terms that refer to the NKs of a cognitive discipline will tend to be *projectible* (in Nelson Goodman’s sense (1954). Thus, the cognitive disciplines that contain terms of NKs tend to make *correct predictions*.

To be clear, we do not contend that the previous epistemic characteristics are either necessary or sufficient epistemic criteria for functional cognitive kindhood. Nevertheless, they are typically present in a theoretical description of a system that is structured in a causal functional manner characteristic of CF-NKs.

To summarize, what we have been articulating here is an approach to conceptualizing the parts of cognitive science that irreducibly refer to natural kinds of cognitive capacities, including those that are in the interface of cognitive psychology and neuroscience.

## Cognitive Natural Kinds, Individual Variation, and Multiple Realization

There are multiple ways in which philosophers and other researchers have understood both variation and multiple realization (e.g., Aizawa, 2017, Figdor, 2010). These are phenomena that frequently occur in psychology and cognitive science, but also in many branches of biology, e.g., evolutionary developmental biology (see, e.g., Abouheif, 1997). Here we will use these terms in ways that are more or less in tune with the way in which they are used in these special sciences.

In biology, a usual manner of understanding variation starts out by distinguishing intraspecies from interspecies variation. Intraspecies variation occurs when a certain character that is typically present in the members of a species is differently instantiated in different individuals of the species. The nomological possibility of the existence of variation of a character present in a species is called “variability.” Interspecies variation of a character that occurs usually in different but closely related species also involves the different instantiation of that character in typical members of the different species. Here we will talk mostly about intraspecies cognitive variation, but it is important to understand that interspecies variation involves different concepts than intraspecies variation (e.g., the biological and functional concepts of homology, convergence, etc.).^29^

Concerning CF-systems, the definition of intraspecies cognitive variation is very similar to its counterpart in biology: given a set of CF-systems belonging to the same NK, call it NK* in species *H*, and a cognitive character *C* that most of these CF-systems share,^30^ we say that *C* exhibits *variation* in NK* of species *H* with respect to *C* whenever *C* exhibits some differences in at least some of the token CF-systems of NK*.

On the other hand, multiple realization, as we understand it, stands for a relationship between a kind (of capacity, character, etc.) and the implementation(s) of its tokens at lower levels of organization: a kind *K* belonging to a discipline at level *n* is multiply realized at level *n-m* (where n > m > 1) when there are two or more non-intersecting subsets of tokens of kind *K* (call these subsets *K*_*1*_, …, *K*_*p*_) such that the implementation of the tokens of any subset *K*_*i*_ of *K* is of a different kind than the implementation of the tokens of any other subset *K*_*j*_ of *K* in a discipline at level *n-m*.^31^ Thus, *multiple realization* of a *cognitive* capacity CF-NK will occur when non-intersecting subsets of members of that kind are implemented by different kinds belonging to a lower level of organization –whether neurological, molecular, genetic, etc.^32^

A few very important points to emphasize. First, we do not claim that either intraspecies variation or multiple realization are phenomena that necessarily occur in the special sciences. That is, it is not the case that, as a matter of nomological, metaphysical or logical necessity, all CF-NKs are multiply realized.^33^ We find no convincing argument to this effect. It is rather a matter of empirical fact that individual intraspecies variations sometimes occur for different causes.^34^ Our view is that multiple realization sometimes occurs in the biological and psychological worlds, so to speak, as a matter of contingent fact.

However, second, it is an advantage of our view that it allows for the presence of some cognitive variation and multiple realization in our CF-systems and CF-NK. There is simply no doubt concerning the existence of intraspecies variation in cognitive science, and mounting evidence that multiple realization may also be an important phenomenon in this field. Furthermore, other yet unknown types of multiple realization should not be ruled out a priori.

As we can see, our view allows for the presence of some cognitive variation in the instances of a CF-NK, i.e., it allows variations in the tokens of a CF-NK (of different individuals in a population or species); say, possibly, working memory, selective attention, episodic memory, etc. This is because, in our own characterization, the different tokens of a CF-NK *tend* to (but need not) have all of the cognitive characters that constitute the CF-NK (either the same kinds of CF-subsystems, or the same kinds of interactions two or more of these subsystems exhibit).^35^

Multiple realization poses different questions for our view. When the same CF-NK, say NK*, appearing in two or more populations of the same species (or in two different species) is implemented by two (or more) distinct kinds of neural systems or networks, then we shall say that NK* is multiply realized in those populations. But what shall we say about NK*; that it is one or *two distinct* kinds?^36^

In our view, there are two different ways in which these questions can be answered depending upon whether we are talking about a simple or a complex CF-NK. In the case of cognitively complex NKs, multiple realization may occur when the members of a CF-NK in the same species or population *vary* because they have as parts some CF-subsystems that are distinct in kind. For example, when one CF-system *T*_*1*_ belonging to CF-NK* has CF-subsystems of *kinds S*_*1*_, *S*_*2*_, *S*_*3*_ and *S*_*4*_ as parts, while another member *T*_*2*_ of CF-NK* has members of kinds *S*_*1*_, *S*_*3*_ and *S*_*4*_ only –where both still count as members of CF-NK*. Thus, in this case, it is to be expected that the implementation of *T*_*1*_ will differ from that of *T*_*2*_. Therefore, NK* is multiply realized.

A second case of multiple realization could in principle occur when we are looking at a *simple* CF-NK, e.g., when two distinct *kinds* of neural systems implement a simple CF-NK. What shall we do with this case?^37^ Of course, *ex hypothesi* at the *neural* level this CF-NK would correspond to two distinct neural kinds. The question is whether this entails that the CF-NK in fact consists of distinct *cognitive* kinds; *cognitively*, its members would perform the same function and even their functional role in other complex systems may be the same in each case. Piccinini & Craver’s (2011) formerly held the view that a splitting strategy was mandatory in these cases. By “splitting strategy” they mean the procedure by which the simple NK in fact would be, in cognitive terms, two distinct kinds.

For example, a classical mirror neuron appears to implement a simple CF-NK that associates sensory and motor information concerning the same specific “action”. To us, this appears to be something genuinely like a single simple cognitive NK. Can there be two neurological kinds of neurons with the same cognitive classical mirror function (i.e., that of associating sensory and motor information concerning the same specific “action”)? And if this were to occur (something not at all improbable), shall we say they are two cognitive kinds, even though they perform the same cognitive feat? We do not think so. As a matter of fact, it has been shown –see e.g., Aizawa (2017), Abouheif (1997)– that this is contrary to scientific practice.^38^

In Piccinini and Craver’s former view (2011) scientists *should always take* the “splitting strategy”, but in our view this claim can only be supported if one accepts certain a priori normative assumptions about science that we reject. The CF-systems approach to cognitive capacities that we present here allows us to accommodate alternative answers to some old problems in psychiatry. Since the development and subsequent improvements of the main classificatory systems of mental disorders (e.g., The Diagnostic and Statistical Manual of Mental Disorders (DSM-III and subsequent), published by the American Psychiatric Association (APA)) a couple of features have been of concern to the discipline, namely, within-category heterogeneity and comorbidity. These features, shared by most of the psychiatric taxonomies that have been proposed to date, have for a long time been considered serious problems that appear to be impervious to a solution. Within-category heterogeneity occurs when the category that is used to refer to a mental disorder covers cases whose symptoms are very heterogeneous and are not necessarily present in all cases of the disorder. Further, comorbidity is the coexistence of symptoms of two or more psychiatric disorders in a single patient. Usually, both features are attributed to taxonomic deficiencies.

The view that we defend in Salcedo-Gómez & García (2023) takes some of the claims that aredeveloped here to argue that these features are not necessarily indications of defective classifications: they are results of the actual human cognitive organization, taken as a whole. Such organization has, in our view, three characteristics that explain why many psychiatric disorders –understood as dysfunctions of one or more CF-systems– are either comorbid and/or internally heterogeneous. First, many underlying cognitive capacities usually are causally interconnected in regular but very complex ways so that a cognitive dysfunction in one CF-system of kind *K* tends to result in cognitive dysfunctions in CF-systems of other kinds. Second, there are individual cognitive variations in the manner of operation of any given capacity of the same kind (CF-NK) in different individuals, in a way that dysfunctions of the same kind of CF-system in two or more individuals may result in different cognitive symptoms. And third, the neurological implementation of most CF-NKs is and can be multiply realized in some cases, and when this occurs it may have as a result the heterogeneity observed in their dysfunctions at the cognitive level.

## Functionalism, Mechanism, and the Neuro-Cognitive Debate

Some of the important issues we will now examine have already been foreshadowed in the previous section. These are the following:

1. Is psychology autonomous from neuroscience? In what respect, to what degree, and why?
2. Should all psychological explanations be integrated with neuroscientific explanations?
3. How do these two different types of research constrain each other?

In this debate, there are roughly two positions: the mechanistic position and the functionalist position. Functionalists tend to argue that psychology is, at least, taxonomically autonomous from neural science (Weiskopf, 2017; Roth & Cummins, 2017), while mechanists tend to deny this (Piccinini & Craver, 2011; Boone & Piccinini, 2016). Both are influenced by the work of Cummins (1975); hence the debate is framed in the context of explaining cognitive capacities. Although it is a debate in the philosophy of cognitive science, it also reflects methodological standpoints often adopted by cognitive scientists themselves. We will describe them in broad terms.

The mechanistic position (e.g. Boone & Piccinini, 2016, Craver, 2001, Miłkowski, 2016) contends that genuine cognitive explanations need to be presented in terms of mechanisms, where this term is understood as developed by the New Mechanical Philosophy program in the philosophy of science. Within this framework, a mechanism is a system that produces regular activity through the interaction of its parts, where parts are understood to be spatiotemporally located entities within the boundaries of a phenomenon (see Glennan, 2017, ch. 2). According to this view, scientific research is performed by spatiotemporally^39^ decomposing a system up to the point where it can be described as an interaction of its ultimately constituent parts.

For Piccinini and Craver (2011), functional explanations at the level of psychology are considered to be mechanism sketches. These sketches may be helpful in the search of complete explanations at the level of neuroscience. Psychological explanations are provisional until validated by further decomposition of mechanisms at the level of neuroscience. When psychological kinds are found to be realized by multiple mechanisms, mechanists advocate a splitting strategy. What is deemed to be a single cognitive kind in the mechanism sketch appears as two different kinds in the putative complete explanation. Apparent autonomy at the level of psychology is merely a product of incomplete information. A multiply realized cognitive kind is not yet scientifically respectable.

Recently, defenders of the mechanist position have softened their views in important ways. First, by allowing that complete mechanical explanations need not end in an account at the neural level, but at the level of the mathematical modeling of the interaction of large groups of neurons, such as exemplified in computational neuroscience. Second, by allowing that explanations need not “split” up to the point of single types of mechanisms, but classes of similar mechanisms. This allows for individual differences in implementation but only up to the degree of mechanistic abstraction. Mechanists are insistent that this does not amount to accepting multiple realization outside this case (e.g., Boone & Piccinini, 2016, p. 688). Although we find this answer unsatisfying, we do not have the necessary space here to develop an argument to show why the appeal to “abstraction” by Piccinini and Boone in this context does not support the idea that these are cases where there are “similar implementational mechanisms” which do not amount to multiple realization.^40^

By contrast, defenders of functionalism in the sense under discussion (e.g., Weiskopf, 2017, Shapiro, 2019, Roth & Cummins, 2017) contend that psychological explanations are autonomous. Although parts of psychology may be integrated with neuroscience, psychological explanations and taxonomies can be complete on their own. Thus, cognitive kinds need not have a type-correspondence to neuroscientific kinds. Just like for mechanists, psychological kinds are individuated according to causal contributions of the functions performed by the capacity under study, but models need not bottom out (i.e., correspond in a one to one manner) in terms of mechanistic explanations at the level of neuroscience. Psychological kinds may be robust in their own way.

We contend that functionalism is a better description of working cognitive scientists, and that it is not committed to the idea that robust psychological kinds will correspond to kinds mechanically individuated at the level of neuroscience. In our view, this is an empirical matter that cannot be decided on conceptual grounds. We contend that our functionalism is compatible with the idea that there may be more than one neuroscientific explanations of the same kind of cognitive capacity (CF-NK), and that when this occurs, the resulting neuroscientific explanations do not necessarily lack theoretical interest. The two resulting explanations may be illuminating and interesting even though there is no one to one correspondence between the underlying two kinds of phenomena.

Furthermore, as we already explained, we accept the idea that there likely are kinds of cognitive capacities that are *natural*. In contrast, we think that most present-day functionalists have not provided a framework that allows them to distinguish between merely instrumental theoretical constructs, which are empirically adequate and serve predicting purposes, from those that represent the actual cognitive capacities of the system under study. Without much explicit philosophical work on this issue, functionalists are not able to answer the realist’s concerns.

Our position aims to fill this gap. A functionalist who claims that psychology is a science taxonomically autonomous from neuroscience will be required to have good reasons to think that at least some of the central kinds of entities postulated in psychology can have a strong ontological status –lest she be subject to the objection that psychological entities are useful heuristic devices having no ontological import (e.g., Dennett, 1987, Churchland, 1984, Stich, 1983, and Schiffer, 1987, to name just a few).^41^

This is not only a matter of metaphysics, but also of methodology. For example, some constructs with a high degree of idealization (e.g. Weiskopf, 2017) may pose a problem for a realist about cognitive capacities. Also, if empirical robustness is the only criterion, we should note that there are robust phenomena that are products of statistical artifacts, such as correlations, or context dependent or culturally bound phenomena that should not count as cognitive capacities and are not considered as such by working cognitive psychologists and cognitive neuroscientists. Without a principled way to make a distinction between cognitive natural kinds and other categories, this important difference between actual capacities and other cognitive phenomena would merely rest in an unsuitable form of scientific conventionalism.

Finally, concerning CF-systems and their kinds, there are two related questions that are nonetheless distinct. First, an ontological question concerning which entities, activities, and causal interactions constitute a CF- system and which ones do not. Second, an epistemological question concerning how we know or find out which entities and activities, etc., constitute or are parts of this or that system. Carl Craver and others (Craver et al., 2021) recently wrote a paper addressing the epistemological question. It is a very interesting paper that shows how to use the epistemic tools of an interventionist view of causation to answer it (see also Shapiro, 2019). Concerning the ontological question, much can be said about it. Nevertheless, this is something that falls outside the scope and limits of this paper.

## Concluding Remarks

Given the limitations inherent to this publication format, here we can only develop the central tenets of our view. Since it consists in a complex attempt to interweave views from a variety of sources, both philosophical and cognitive, it is an attempt to legitimize a form of making cognitive science that is at least partially based on the postulation of functional systems to explain (human and/or nonhuman) cognitive capacities, and to defend a form of autonomy of the cognitive with respect to the neural. We also consider a number of important objections to it, by articulating the idea that there is a functional manner of looking at parts of cognition that can be conceptualized as some form of natural kind.

Furthermore, we think that a taxonomy based upon cognitive-functional principles is autonomous from other forms of taxonomizing, for example, a neurobiological, a cellular or a molecular taxonomies. This is because, as we have contended, *pace* Piccinini and Craver (2011) there are no a priori grounds to suspect that all cognitive functional organization fits with neurobiological organization into a common type. Nevertheless, in contrast to previous works which have defended taxonomical autonomy (e.g. Weiskopf, 2017), we do not hold that psychology is explanatorily autonomous. This is because psychological kinds exhibit a nested structure, with simple cognitive kinds located at the foundational level. The functional organization of simple cognitive kinds is (at least) partially constrained by the causal profile of the neurobiological kinds that embody them, highlighting the need for interdisciplinary integration to achieve a comprehensive psychological explanation at this level. However, it is unwarranted to assume a continuous dependence on neurobiology in the progression towards complex cognitive phenomena; as functional organization, exhibiting its own causal profile, is not exclusively dictated by regularities at the neurobiological level.

## Data Availability

No datasets were generated or analysed during the current study.

## Footnotes

1 Cummins’ notion of a functional system is explained in more detail in Sect. 3. As we shall see, understood in this broad manner, functional processing is not necessarily married to any “representational” or symbolic hypothesis, neither does it entail a form of connexionism or of Bayesianism in itself. And it remains to be seen to what extent a broad functionalism of the sort we defend here is compatible with embodied and/or situational modeling.

2 Note that our view makes no commitment one way or the other to the explanations and entities that are postulated in so-called folk psychology.

3 The relation of cognitive neuroscience with cognitive psychology has been varied. Cognitive neuroscience emerged in the 1970s, differentiating itself from earlier neuroanatomy and neurophysiology research. It integrated with cognitive science and aligned with cognitive psychology and neuropsychological methods for studying behavioral dissociations in brain-damaged subjects (Roskies, 2021). The rapid development of imaging technologies in the late 1980s provided tools to search for neural correlations of psychological functions. Sometimes neuroscientific explanations of cognitive phenomena are partially integrated or seemingly could be integrated in principle with psychological models. However, many cognitive capacities remain difficult to study through neuroanatomical correlation due to the complex interactions between multiple regions. While brain regional functions are still studied in cognitive neuroscience, other explanations often exceed traditional cognitive descriptions. A great number of fruitful and empirically adequate psychological models so far remain far from being integrated into neuroscientific research, such as Baddeley’s (2000) model for working memory (Weiskopf, 2017).

4 We are not saying that all of the kinds of functional capacities that have been postulated in cognitive science are natural kinds in our sense –some of them may lack the characteristics necessary to be what we call a “cognitive functional natural kind”.

5 Here we will use “nomological” in a broader sense than usual to include the existence of simple or complex regular causal dispositions of a class of entities as part of the furniture of the World that is “nomological”–where the existence of dispositions does not imply the existence of “Laws”.

6 The initial proposal is due to philosopher Cummins (1975).

7 The notion of *part* that is required by our characterization of a functional system (i.e., the notion that would allow us to say that a certain functional system is a part of another functional system) is not based upon spatiotemporal contiguity. See Craver and Tabery (2015 § 2.2) for a discussion of what may be involved in the notion of part required both for mechanisms and for our functional systems.

8 More precisely, we have a system *S* that is composed of other systems, *S*_1_, …, *S*_n_. *S* has the *disposition* to do *ψ* in virtue of *S*_1_’s, …, *S*_n_’s *dispositions* to do *φ*1, …, *φ*_n_, respectively. When such conditions are met, we shall say that *the functions of S*_*1*_, *…*,* Sn* are *φ1*, …, *φn*, respectively, relative to the larger context of *S*’s disposition to *ψ* – such that *φ*_n_ ≠ *ψ*.

9 A preliminary development of some of these ideas can be found in the literature on cognitive architecture without mention of the metaphysics of natural kinds (see e.g., Carruthers, 2005; Sperber, 2001). Here we do not assume that CF-systems need to be vastly modular; however, the more modular a system is, the easier it is to maintain its functional integrity in evolution and to a causal interventionist treatment of its cognitive organization.

10 Elsewhere, García (2014) has argued that the notion of function that is most relevant to talk about CF-systems and their kinds, the variations and homology relations among kinds of CF-systems is Cummins’ notion –the so-called “systems-analytical notion of function.”

11 In philosophy of psychology and biology it is commonly accepted that some cognitive and biological systems are *multiply realized* at lower levels. See Striedter and Northcutt (1991), Abouheif (1997) in different areas of biology; Aizawa (2017) in vision theory; and Figdor (2010) in the philosophy of cognitive science.

12 We are using this example of CF-analysis in cognitive psychology as an illustration of the kinds of functional organizations that are oftentimes postulated in this discipline. However, this should not be taken as an endorsement of Baddeley’s particular CF-analysis of working memory. Furthermore, there are many other examples of this sort, e.g., Conway’s CF-analyses of episodic memory (2005) and Hassabis & Maguire’s functional analysis of autobiographical memory (2007). Weiskopf (2017, 64–65) also uses the example of WM to illustrate a similar point.

13 We shall have more to say about this issue further below.

14 As we will show in Sect. “Cognitive Functional Natural Kinds (CF-NK)”, the simple/complex distinction as applied to CF-systems (and kinds of them) is also crucial to conceptualize a notion of multiple realization.

15 Wimsatt (2013) discusses with some detail the different factors that may be involved in the evolutionary stability and robustness and in the changes of –both biological and cognitive– functional organizations. He argues that the robustness of a functional organization does not always result in a decrease of its evolvability.

16 Wagner and Schwenk (2000) argue that the constraints that the functional organization of a functional system of a kind exerts over the possible non-lethal variations that can arise in the kind, are evolutionary constraints themselves. They call it “functional integrity”.

17 In evolutionary biology, the evolvability of a character in an organism of a certain kind is a function of “the organism’s capacity to facilitate the generation of non-lethal selectable phenotypic variation from random mutation” (Kirschner, M., & Gerhart, J. (1998); also see García, 2007, p. 63). Since, in this context, the notion of evolvability and that of variation are closely related, in order to be able to speak of the evolvability of a cognitive capacity (understood as a CF-system), we will need to make use of notions of *functional variation* and also (as García (2010) explains) of functional homology–where the latter is understood as the presence of the same kind of functional system in a lineage, due to descendance from the last common ancestor. The articulation of these notions is accomplished in García (2010, 2014). However, we do not assume that all the natural kinds of CF systems (CF-NKs) are necessarily the result of evolutionary taxonomizing (e.g., complex CF-NKs that are mostly a result of human culture and not biological evolution). In these cases, we can talk about variation but not about homology and/or evolvability in the strictest biological sense.

18 As we shall see, this consequence (along with others) is what allows for the existence of *variation* and *multiple realization* inside a class of CF-systems that form a CF- NK.

19 Some, but not most, of these systems may also belong to more than one CF-natural kind. Furthermore, neither our CF-NKs nor their functional subsystems need to be understood as “modules” or as being encapsulated –although some may be.

20 One of the possible ways in which multiple realization of cognitive natural kinds can occur is here, where token simple CF-systems get to be explained at a lower level of organization. See further below in the next section.

21 The extent to which the *S*_*i*_ of *S* are differently organized causally is a contingent question to be studied in an a posteriori manner. Normally, the differences in functional organization are not great.

22 Note that one corollary of our view of CF-NK is that if S_1_ is a component CF-subsystem of S_2_, and S_2_ belongs to a CF-NK, then it is probable that S_1_ also belongs to (another) CF-NK.

23 This is where something like Boyd’s notion of natural kind enters the scene, since he was interested in fashioning a concept of natural kind that can actually be applied to types of biological and psychological phenomena (e.g., organisms of different types, their physiology, morphology, environments, their changes in time, their interactions, their cognitive capacities, and the similarities and differences in all these respects).

24 For example, Woodward argues that many of the causal structures postulated by dynamical systems theory may not be subject to an interventionist treatment such as his.

25 Shapiro (2019) convincingly argues that functionalism –as well as mechanism– could unproblematically involve a notion of causation that is interventionist, such as Woodward’s.

26 The Boydean view that initially inspired us in the articulation of our natural kind concept as applied to cognitive capacities is the one he defends in 1999 and 2010. In one of his latest writings, published in Boyd (2021), he argues that it makes a lot of sense to speak of natural kinds in connection with the signals that many nonhuman animals use primarily for communicative purposes (see 2021, 2886). Since the notion of a natural kind that we propose here is intended for the purpose of distinguishing those (human) concepts, and the scientific theories that accompany them, that have a certain theoretical and ontological preferred status from other concepts that may at most be heuristically fruitful yet not to be taken seriously from an ontic scientific perspective, a view like the one Boyd presents in 2021 does not seem to fit the bill.

27 We want to distinguish the study of cognitive capacities from the –also legitimate– study of other context-dependent cognitive processes which also exhibit a causal, hierarchical structure. As an example of this, we can think of many of the constructs proposed in social psychology that are not bona fide cognitive capacities e.g. *inquisitive curiosity* (Iurino et al., 2018). This goes in line with Khalidi (2015, 1391) stressing how his view allows for natural kinds in social and artifactual realms. CF-NKs provide normative criteria, consistent with scientific practice, to characterize cognitive natural kinds in a way that differentiates them from other kinds related to cognitive endeavors which do not describe cognitive capacities.

28 We want to distinguish the study of cognitive capacities from the –also legitimate– study of other context-dependent cognitive processes which also exhibit a causal, hierarchical structure. As an example of this, we can think of many of the constructs proposed in social psychology that are not bona fide cognitive capacities e.g. *inquisitive curiosity* (Iurino et al., 2018). This goes in line with Khalidi (2015, 1391) stressing how his view allows for natural kinds in social and artifactual realms. CF-NKs provide normative criteria, consistent with scientific practice, to characterize cognitive natural kinds in a way that differentiates them from other kinds related to cognitive endeavors which do not describe cognitive capacities.

29 In cognitive psychology, intraspecies variation is called *individual difference* and is used to talk about variation within the same species, (specifically humans). On the other hand, interspecies variation is used mostly in comparative psychology; as when a researcher is studying the different ways in which working memory respectively operates in humans and chimpanzees –much of what is involved in this form of variation is discussed in García’s papers (2010, 2014).

30 By “cognitive character” we mean things like the kinds of CF-subsystems that a CF-system has as parts, the kinds of interactions two or more of these subsystems exhibit, the kinds of cognitive outcome the CF-system typically produces or should produce, the kinds of information or representation that it accepts as inputs or that it gives as outputs, etc.

31 Thus, as we understand it, multiple realization is a relationship between kinds belonging to different disciplinary levels, while variation is not. For a detailed discussion of other ways in which multiple realization is treated in the special sciences, see Aizawa, 2017.

32 Thus, as we understand implementation, it is sometimes a one-to-many relationship between kinds belonging to different levels of organization (but not necessarily so). Furthermore, it is an explanatory relationship between different-level kinds: reference to each of the multiple lower-level kinds explains how some (but not all) of the tokens of the higher-level kind perform their functions.

33 The same is true in biology, there may be various somewhat distinct regulatory gene networks (RGNs) that have as a consequence the development of non-webbed digits in the same or closely related species of vertebrates –yet biologists think of them as the same kind of developmental pathway having the same developmental function. Usually, the differences at a molecular level are not great; only portions of the RGN are distinct. Yet, these differences are not causally irrelevant in each case; they are necessary for the corresponding digit development.

34 Some biologists think variations are sometimes the result of the manner in which ontogenetic development in many living beings is structured through evolution–but none of them claim that this would be the only causal scenario responsible for variation. Mutations and other phenomena also bear some responsibility.

35 For example, within certain limits, working memory (WM) can be more “effective” (comprise more items of information, operate faster, etc.) in different individuals of a human population and still count as WM. Further, its tokens tend to be composed of the same CF-subsystems, but there can be individual variations in this respect that can still count as tokens of *that* CF-NK.

36 Figdor (2010) explores some of the large problems that arise for the special sciences –particularly for the relations between cognitive science and the neurosciences –in connection with the phenomenon of multiple realization.

37 Cognitive neuroscientists would probably choose what Craver (2009, 581) calls the “*splitting strategy”*: to conclude that the simple NK in fact would be, in cognitive terms, *two distinct kinds*, two CF-NKs. But this is so only because most cognitive neuroscientists implicitly or explicitly accept some generalized form of reductionism; something that is very controversial.

38 Aizawa (2017) convincingly argues that what Craver calls the “splitting strategy” is not the only strategy adopted in, e.g., vision science. In this discipline, there are a number of cases where two or more distinct kinds of phenomena implement a single kind of phenomenon at a higher level of organization. Oftentimes, the higher-level phenomenon continues to be considered as a single kind in spite of its known multiple realizations. Other cases where the splitting strategy is not adopted are described in Abouheif (1997) in the field of molecular developmental genetics.

39 Whether this decomposition also is, or only is, functional, according to the New Mechanists, is not clear. The answer (when any) depends upon the mechanist one is talking with. Sometimes someone like Craver seems to accept a partial functionalist answer, but in general, functionalism is not an essential component of mechanism. Furthermore, it appears that, by their very characterization, mechanisms have (one or more) spatial localizations. The entities that constitute a mechanism may not be spatially contiguous to each other – i.e. they may be all over the place in, say, the brain– and yet they have some localization of sorts. In contrast, CF-systems are individuated by their cognitive function (not localization); and although *token* CF-systems of the same CF-NK are localized somewhere in an organism, not all tokens are necessarily in the same type of place(s).

40 We agree with Boone and Piccinini (2016) that there are cases where the differences in the underlying implementational mechanisms may be very small. They may even constitute the majority of cases of multiple realization in biology and in cognitive science. However, there are also cases in cognitive science that consist in the presence of differences that cannot under any criterion be classified as “very small”. See Figdor (2010). But even in the cases of “very small” differences, the differences in question are explanatorily and causally relevant. So, Boone and Piccinini at the very least must acknowledge this is a controversial issue in cognitive science.

41 In the 1980’s there was a movement among philosophers of mind and language in the analytic tradition to adopt positions that asserted that, for various philosophical reasons, there were no mental or intentional entities and processes (including psychological and/or folk psychological entities) understood functionally, that their existence could not be the subject of a serious science, and that at most these entities had some heuristic value in our everyday dealings with the world.
